# 二线及以上应用贝伐珠单抗联合化疗治疗晚期非鳞非小细胞肺癌的疗效及安全性观察

**DOI:** 10.3779/j.issn.1009-3419.2018.07.02

**Published:** 2018-07-20

**Authors:** 宣轩 郑, 慧娟 王, 国伟 张, 相涛 闫, 智勇 马

**Affiliations:** 450008 郑州，郑州大学附属肿瘤医院呼吸内科 Department of Respiratory Medicine, the Affiliated Cancer Hospital of Zhengzhou University, Henan Cancer Hospital, Zhengzhou 450008, China

**Keywords:** 贝伐珠单抗, 肺肿瘤, 化疗, 疗效, 脑转移, Bevacizumab, Lung neoplasms, Chemotherapy, Efficacy, Brain metastases

## Abstract

**背景与目的:**

贝伐珠单抗联合含铂双药化疗被推荐为无驱动基因的晚期非鳞非小细胞肺癌（non-small cell lung cancer, NSCLC）患者的一线治疗方案，但此方案用于二线及以上非鳞NSCLC的研究并不普遍。本研究拟探讨二线及以上应用贝伐珠单抗联合化疗治疗晚期非鳞NSCLC的疗效和安全性。

**方法:**

回顾性分析郑州大学附属肿瘤医院2014年1月-2017年6月间一线治疗进展后应用贝伐珠单抗的晚期非鳞NSCLC患者的临床资料，采用*Kaplan-Meier*法、*Log-rank*检验和*Cox*模型进行统计分析。

**结果:**

这项研究共纳入62例患者，总体的客观缓解率（objective response rate, ORR）为32.2%，疾病控制率（disease control rate, DCR）为96.8%。中位无进展生存期（progression-free survival, PFS）为6.4个月（95%CI: 6.05-6.83），中位总生存期（overall survival, OS）为20.4个月（95%CI: 12.98-27.76）。在亚组分析中，脑转移患者与无脑转移患者的中位PFS差异无统计学意义（6.2个月*vs* 6.4个月，*P*=0.052）。贝伐珠单抗的应用周期（> 6个或≤6个）是PFS的独立影响因素（*P*=0.004）。最常见的不良反应有白细胞减少、乏力、恶心、血小板减少和高血压。

**结论:**

二线及以上应用贝伐珠单抗联合化疗治疗晚期非鳞NSCLC的疗效显著且安全性良好。

## Abstract (en)

**Background and objective:**

Bevacizumab combined with platinum-based chemotherapy has been recommended as the first-line agent in advanced nonsquamous non-small cell lung cancer (NSCLC) without driven gene, but this regimen is not common in the second-line or later-line treatment of non-squamous NSCLC. The aim of this study is to investigate the efficacy and safety of bevacizumab combined with chemotherapy as second-line or later-line treatment in advanced non-squamous NSCLC.

**Methods:**

We retrospectively reviewed the clinical data of advanced nonsquamous NSCLC patients who were treated with bevacizumab after first-line treatment failure and they were hospitalized in the Affiliated Cancer Hospital of Zhengzhou University from January 2014 to June 2017, and *Kaplan-Meier* method, *Log-rank* test and *Cox* model were used for analysis.

**Results:**

A total of 62 patients were included in the analysis. The total objective response rate (ORR) was 32.2%, and the disease control rate (DCR) was 96.8%. The median progression-free survival (PFS) was 6.4 months (95%CI: 6.05-6.83), and the median overall survival (OS) was 20.4 months (95%CI: 12.98-27.76). In the subgroup analysis, there was no significant difference in median PFS between patients with brain metastases and those without brain metastases (6.2 months *vs* 6.4 months, *P*=0.052). Cycles of bevacizumab (> 6 or ≤6 cycles) was an independent influencing factor of PFS (*P*=0.004). The most common adverse events were leukopenia, fatigue, nausea, thrombocytopenia and hypertension.

**Conclusion:**

In the second-line or later-line treatment, bevacizumab combined with chemotherapy is an effective and safe regimen for advanced non-squamous NSCLC.

肺癌是全球高发病率、高死亡率的疾病^[1]^，其中非小细胞肺癌（non-small cell lung cancer, NSCLC）约占肺癌的85%，且56%的患者确诊时已发生转移^[2]^。晚期NSCLC的一线治疗多应用靶向治疗或含铂双药化疗，然而，对于靶向治疗后耐药和化疗后进展的晚期NSCLC，二线及以上应用化疗的疗效较差，总生存期不超过10个月^[3]^。随着免疫抑制剂nivolumab、pembrolizumab和atezolizumab的出现，二线及以上NSCLC的治疗有了新选择，但是，免疫抑制剂有效率低、价格昂贵。如何提高治疗后进展的晚期NSCLC的疗效是目前治疗的热点。

抗血管生成药物是晚期NSCLC有效的治疗策略。在NSCLC患者中，血管内皮生长因子（vascular endothelial growth factor, VEGF）高表达与诱导血管形成、预后不良和淋巴结转移显著相关^[4, 5]^。此外，Chen等^[6]^发现晚期NSCLC患者肿瘤标本的微血管密度（血管生成的间接测量指标）高于早期患者，淋巴结转移患者的微血管密度也高于未转移患者。基于这一理论，抗血管生成可达到抗肿瘤的目的。抗血管生成药物在NSCLC的治疗中成绩斐然。目前用于治疗NSCLC的抗血管生成药物主要包括贝伐珠单抗^[7-9]^、雷莫芦单抗^[10]^、阿帕替尼^[11]^、重组人血管内皮抑素（恩度）^[12]^，其中贝伐珠单抗的应用尤为普遍。

贝伐珠单抗是一种重组人源化IgG1抗体，通过与VEGF结合而抑制VEGF与血管内皮生长因子受体（vascular endothelial growth factor receptor, VEGFR）的结合，进而抑制肿瘤血管的生成，切断肿瘤的营养供应，达到控制肿瘤的目的，其与化疗的联合能提高化疗的疗效。贝伐珠单抗对多种肿瘤（例如非鳞NSCLC^[7-9]^、结肠癌^[13]^和乳腺癌^[14]^等）均有较好疗效，尤其是非鳞NSCLC。贝伐珠单抗联合含铂双药化疗被推荐为无驱动基因的晚期非鳞NSCLC患者的一线治疗方案，但用于二线及以上的临床研究很少。本研究拟探讨二线及以上应用贝伐珠单抗联合化疗治疗晚期非鳞NSCLC的疗效和安全性。

## 1 资料和方法

### 1.1 一般资料

回顾性分析郑州大学附属肿瘤医院2014年1月-2017年6月间一线治疗进展后应用贝伐珠单抗的晚期非鳞NSCLC患者的临床资料，纳入标准：①组织学分型为非鳞NSCLC；②临床分期为Ⅲb期或Ⅳ期，且一线治疗后进展；③美国东部肿瘤协作组活动状态评分为0分或1分，年龄≥18岁；④根据实体瘤疗效评价标准1.1（Response Evaluation Criteria in Solid Tumors, RECIST 1.1）进行评价，至少有1个可测量病灶；⑤贝伐珠单抗按7.5 mg/kg的常规剂量在化疗第1天给予，其他化疗药物按常规推荐剂量给予，且贝伐珠单抗应用≥2个周期，治疗方案应用直至进展或不能耐受不良反应；⑥治疗期间至少2个周期进行1次影像学评估[胸部计算机断层扫描（computed tomography, CT）加或不加头颅磁共振成像（magnetic resonance imaging, MRI）]；⑦可规律进行随访。

### 1.2 研究方法

#### 1.2.1 近期疗效观察

应用贝伐珠单抗联合化疗2个周期后评价，根据RECIST 1.1分为完全缓解（complete response, CR）、部分缓解（partial response, PR）、疾病稳定（stable disease, SD）和疾病进展（progressive disease, PD）。客观缓解率（objective response rate, ORR）=（CR+PR）/（CR+PR+SD+PD）×100%；疾病控制率（disease control rate, DCR）=（CR+PR+SD）/（CR+PR+SD+PD）×100%。

#### 1.2.2 随访和生存分析

釆用电话和门诊方式进行随访，末次随访时间为2018年1月31日。随访时间为3.7个月-28.0个月，中位随访时间14.7个月，无失访患者。无进展生存期（progression-free survival, PFS）定义为患者应用贝伐珠单抗开始至明确为PD的时间；总生存期（overall survival, OS）为患者应用贝伐珠单抗开始至死亡或末次随访的时间。根据患者的年龄、性别、吸烟史、表皮生长因子受体（epidermal growth factor receptor, *EGFR*）基因、间变性淋巴瘤激酶（anaplastic lymphoma kinase, *ALK*）融合蛋白表达、驱动基因（驱动基因阳性即*EGFR*基因或*ALK*融合蛋白表达至少一项阳性，否则为阴性）、脑转移、骨转移、贝伐珠单抗应用周期数（贝伐珠单抗应用 > 6个或≤6个）、治疗方案是否含铂分组，分析与PFS和OS相关的影响因素。

#### 1.2.3 不良反应评价

根据不良事件常用术语标准（Common Terminology Criteria for Adverse Events, CTCAE）4.0版对不良反应进行统计（0级-4级）。

### 1.3 统计学方法

采用SPSS 21.0软件进行统计分析，计数资料的比较采用χ^2^检验或*Fisher*精确检验，生存分析采用*Kaplan-Meier*法和*Log-rank*检验，95%CI的风险比用多因素*Cox*回归分析。检验水准*α*=0.05。

## 2 结果

### 2.1 疗效

经筛选共纳入62例患者，5例患者未观察到疾病进展，PFS删失率为8.06%；17例患者未观察到死亡，OS删失率为27.4%。62例患者的临床特征见表 1。联合贝伐珠单抗的治疗方案包括含铂双药（含铂组）和单药（无铂组），其中含铂方案包括：培美曲塞（24例）、吉西他滨（6例）、多西他赛（5例）、紫杉醇（2例）和长春瑞滨（1例）；不含铂方案包括：培美曲塞（13例）和多西他赛（11例）。

**1:** 患者的临床特征 Clinical characteristics of patients

Category	*n* (%)
Age (yr)	
≥60	18 (29.0)
< 60	44 (71.0)
Gender	
Male	21 (33.9)
Female	41 (66.1)
Smoking status	
Ever	56 (90.3)
Never	6 (9.7)
*EGFR* mutation	
Positive	30 (48.4)
Negative	32 (51.6)
*ALK* rearrangement	
Positive	13 (21.0)
Negative	49 (79.0)
Driver mutation	
Yes	43 (69.4)
No	19 (30.6)
Brain metastases	
Yes	28 (45.2)
No	34 (54.8)
Bone metastases	
Yes	25 (40.3)
No	37 (59.7)
Cycles of bevacizumab	
≤6	32 (51.6)
> 6	30 (48.4)
Platinum-based regimens	
Yes	24 (38.7)
No	38 (61.3)
EGFR: epidermal growth factor receptor; ALK: anaplasticlymphoma kinase.	

62例患者均可评估疗效，其中PR 20例，SD 40例，PD 2例，无CR患者；ORR为32.2%，DCR为96.8%。其中，含铂组与无铂组的ORR值差异无统计学意义（χ^2^=0.409, *P*=0.944）。总体的中位PFS为6.4个月（95%CI: 6.05-6.83），中位OS为20.4个月（95%CI: 12.98-27.76）。多因素分析见表 2。在亚组分析中，脑转移患者与无脑转移患者的中位PFS差异无统计学意义（6.2个月*vs* 6.4个月，HR=0.208，95%CI: 0.492-1.045，*P*=0.052）。贝伐珠单抗应用 > 6个周期患者的中位PFS长于贝伐珠单抗应用≤6个周期患者的中位PFS（9.3个月*vs* 5.4个月），且差异有显著的统计学意义（HR=0.290, 95%CI: 0.124-0.678, *P*=0.004）（图 1）。贝伐珠单抗的应用周期数（> 6个或≤6个）是PFS的独立影响因素。

**2:** *Cox*多因素分析 *Cox* multivariate analysis

Category	PFS			OS	
	HR (95%CI)	*P*		HR (95%CI)	*P*
Age (yr)	1.531 (0.680-3.444)	0.304		0.847 (0.287-2.497)	0.764
Gender	0.376 (0.103-1.310)	0.123		0.565 (0.186-1.713)	0.313
Smoking status	2.806 (0.738-9.665)	0.130		2.350 (0.989-5.871)	0.097
*EGFR* mutation	0.882 (0.134-5.810)	0.896		0.551 (0.059-5.122)	0.600
*ALK* rearrangement	0.941 (0.164-5.388)	0.946		0.889 (0.158-4.984)	0.893
Driver mutation	0.933 (0.136-6.404)	0.943		1.470 (0.167-12.92)	0.728
Brain metastases	0.208 (0.492-1.045)	0.052		0.673 (0.364-1.548)	0.250
Bone metastases	1.593 (0.775-3.274)	0.205		2.540 (0.916-7.042)	0.073
Cycles of bevacizumab	0.290 (0.124-0.678)	0.004		1.297 (0.480-3.504)	0.608
Platinum-based regimens	0.814 (0.389-1.701)	0.584		0.650 (0.272-1.549)	0.331

**1:**
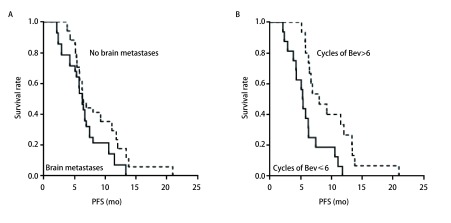
生存曲线。A：有/无脑转移患者的PFS曲线；B：贝伐珠单抗应用 > 6个和≤6个周期患者的PFS曲线。 Survival curve of the patients. A: PFS curves of patients with/without brain metastases; B: PFS curves of patients who used bevacizumab > 6 and ≤6 cycles. Bev: Bevacizumab.

### 2.2 安全性

62例患者均可评估毒副反应，不良反应发生后给予对症处理。最常见的不良反应有白细胞减少、消化道反应、乏力、恶心、血小板减少和高血压。3级-4级不良反应的发生率为32.3%，包括白细胞减少、血小板减少、消化道反应、乏力、高血压（表 3）。

**3:** 治疗相关的的不良反应 Treatment-related adverse events

Adverse events	All grade	Grade 1-2	Grade 3-4
Leukopenia	30	24	6
Thrombocytopenia	12	6	6
Fatigue	26	23	3
Nausea	18	18	0
Vomiting	8	8	0
Decreased appetite	27	23	4
Hypertension	5	4	1
ALT increase	4	4	0
AST increase	6	6	0
Alopecia	2	2	0
Myalgia	6	6	0
Dizziness	2	2	0
ALT: alanine aminotransferase; AST: aspartate aminotransferase.			

## 3 讨论

目前二线及以上应用贝伐珠单抗联合化疗治疗晚期非鳞NSCLC的疗效及安全性的研究屈指可数，本研究发现其疗效引人瞩目，且耐受性良好，在此拟探讨二线及以上应用贝伐珠单抗的近期疗效、脑转移对PFS的影响以及总体中位OS较长的原因。

二线及以上应用贝伐珠单抗治疗晚期NSCLC的其他研究亦表明其疗效显著。ULTIMATE研究^[15]^发现每周紫杉醇联合贝伐珠单抗二、三线治疗晚期非鳞NSCLC患者的ORR为22.5%，中位PFS为5.4个月。另外，Kurishima等^[16]^发现多西他赛联合贝伐珠单抗二、三线治疗NSCLC的ORR和中位PFS分别为26.7%、5.9个月。然而，本研究的ORR高达32.2%、中位PFS长达6.4个月，似乎不亚于上述研究。对于无驱动基因的晚期非鳞NSCLC，培美曲塞或多西他赛单药方案是目前临床实践中常用的二线治疗方案，其ORR约为10%、中位PFS约为3.0个月^[17]^。对比目前临床实践中常用的二线及以上化疗方案，贝伐珠单抗二线及以上治疗晚期非鳞NSCLC的疗效令人惊喜。贝伐珠单抗联合化疗可能是晚期非鳞NSCLC患者二、三线治疗的新选择。

二线以上应用贝伐珠单抗改善了晚期非鳞NSCLC患者的疗效，尤其是脑转移患者。本研究的亚组分析发现脑转移与无脑转移患者的中位PFS无明显差异（6.2个月*vs* 6.4个月，*P*=0.052），中位OS亦无明显差异（18.5个月*vs* 24.5个月，*P*=0.250），这表明贝伐珠单抗联合化疗对晚期非鳞NSCLC脑转移有效。BRAIN研究^[18]^发现二线应用贝伐珠单抗联合厄洛替尼治疗NSCLC脑转移患者的中位PFS为6.3个月，中位OS为12.0个月。本研究与BRAIN研究的中位PFS值相似，贝伐珠单抗联合化疗的疗效似乎毫不逊色。目前脑转移瘤患者常应用放疗，NSCLC脑转移患者接受放疗的中位PFS为3.0个月-3.7个月，中位OS仅为7.4个月-12.2个月，贝伐珠单抗联合化疗的疗效明显优于传统的放疗。另外，本研究患者在应用贝伐珠单抗治疗期间均未出现脑出血，多项研究^[18, 19]^也证明了贝伐珠单抗的治疗未增加脑转移患者发生脑出血的风险。

本研究总体的中位OS长达20.4个月，其中驱动基因阳性与驱动基因阴性患者的中位OS分别为20.4个月、15.2个月，差异无统计学意义（*P*=0.728）。本研究总体的中位OS明显长于ULTIMATE研究^[15]^12.5个月的中位OS，也长于Adjei等^[20]^在贝伐珠单抗联合培美曲塞二线治疗晚期NSCLC研究中观察到的8.6个月的中位OS。本研究中位OS较长的原因考虑可能与EGFR-TKIs或ALK抑制剂的应用相关，其中应用EGFR-TKIs或ALK抑制剂的患者占61.3%。另外，考虑EGFR-TKIs和贝伐珠单抗有协同作用。贝伐珠单抗和EGFR-TKI有共同的c-MET上游通路^[21]^，也许EGFR-TKI治疗后的患者上调了c-MET通路，从而可能导致了VEGF过表达，因此贝伐珠单抗的抗肿瘤疗效得以协同增强。上述研究提示靶向药物的联合应用可能具有协同的抗肿瘤活性，多靶点信号的联合抑制可能代表了第二代靶向治疗的方向，多项临床试验^[22, 23]^的结果令人鼓舞，具体的分子机制尚需要进一步试验证明。

尽管本研究发现贝伐珠单抗联合化疗二线及以上治疗晚期非鳞NSCLC患者的疗效令人惊喜，但是这项研究的局限性亦不可忽视。首先，样本量少。本研究的样本量仅62例，结果不一定客观准确。比如，*Cox*多因素分析发现吸烟史、*EGFR*基因突变、脑转移等的可信区间太宽，考虑与样本量太少相关。其次，此研究为回顾性分析，且患者的治疗方案多样，其结果有待大规模的随机对照试验进一步验证。最后，非一线应用含铂双药联合贝伐珠单抗方案可能会提高ORR、延长中位PFS^[24]^。鉴于此，我们根据治疗方案是否含铂进行了亚组分析。亚组分析显示，含铂组和无铂组的ORR分别为36.8%、25.0%，差异无统计学意义（*χ*^2^=0.409, *P*=0.944）；中位PFS分别为10.6个月、5.7个月，差异亦无统计学意义（*P*=0.584）。根据上述亚组分析暂不考虑含铂双药联合贝伐珠单抗提高ORR、延长中位PFS的可能。

综上所述，二线及以上应用贝伐珠单抗联合化疗治疗晚期非鳞NSCLC的疗效显著，但是，二线及以上应用贝伐珠单抗联合化疗在临床中的使用策略有待进一步研究，如贝伐珠单抗联合哪种化疗方案、长期用药时应用贝伐珠单抗单药还是联合用药等问题。此外也期待更多的研究探讨二线及以上应用贝伐珠单抗联合化疗的疗效及安全性，并进一步完善NSCLC患者治疗的全程管理。

## References

[1] Siegel RL, Miller KD, Jemal A (2018). Cancer statistics, 2018. CA Cancer J Clin.

[2] Ettinger DS, Akerley W, Borghaei H (2012). Non-small cell lung cancer. J Natl Compr Canc Netw.

[3] Lima AB, Macedo LT, Sasse AD (2011). Addition of bevacizumab to chemotherapy in advanced non-small cell lung cancer: a systematic review and *meta*-analysis. PLoS One.

[4] Kojima H, Shijubo N, Yamada G (2005). Clinical significance of vascular endothelial growth factor-C and vascular endothelial growth factor receptor 3 in patients with T1 lung adenocarcinoma. Cancer.

[5] Takizawa H, Kondo K, Fujino H (2006). The balance of VEGF-C and VEGFR-3 mRNA is a predictor of lymph node metastasis in non-small cell lung cancer. Br J Cancer.

[6] Chen ZJ, Le HB, Zhang YK (2009). Microvessel density and expression of thrombospondin-1 in non-small cell lung cancer and their correlation with clinicopathological features. J Int Med Res.

[7] Gautschi O, Rothschild SI, Li Q (2017). Bevacizumab plus pemetrexed versus pemetrexed alone as maintenance therapy for patients with advanced nonsquamous non-small-cell lung cancer: Update From the Swiss Group for Clinical Cancer Research (SAKK) 19/ 09 Trial. Clin Lung Cancer.

[8] Zhou C, Wu YL, Chen G (2015). BEYOND: A randomized, double-blind, placebo-controlled, multicenter, phase Ⅲ study of first-line carboplatin/paclitaxel plus bevacizumab or placebo in chinese patients with advanced or recurrent nonsquamous non-small-cell lung cancer. J Clin Oncol.

[9] Crinò L, Dansin E, Garrido P (2010). Safety and efficacy of first-line bevacizumab-based therapy in advanced non-squamous non-small-cell lung cancer (SAiL, MO19390): A phase 4 study. Lancet Oncol.

[10] Garon EB, Ciuleanu TE, Arrieta O (2014). Ramucirumab plus docetaxel versus placebo plus docetaxel for second-line treatment of stage Ⅳ non-small-cell lung cancer after disease progression on platinum-based therapy (REVEL): a multicentre, double-blind, randomised phase 3 trial. Lancet.

[11] Wang XM, Zhang WH, Du WJ (2017). Efficacy and survival analysis of apatinib in patients with advanced nonsquamous non-small cell lung cancer after failure of first-line treatment. Zhongguo Fei Ai Za Zhi.

[12] Lu S, Li L, Luo Y (2015). A multicenter, open-label, randomized phase Ⅱ controlled study of rh-endostatin (Endostar) in combination with chemotherapy in previously untreated extensive-stage small-cell lung cancer. J Thorac Oncol.

[13] Kawczyk-Krupka A, Sieron-Stoltny K, Latos W (2016). ALA-induced photodynamic effect on vitality, apoptosis, and secretion of vascular endothelial growth factor (VEGF) by colon cancer cells in normoxic enviromment *in vitro*. Photodiagnosis Photodyn Ther.

[14] Zielinski C, Láng I, Inbar M (2016). Bevacizumab plus paclitaxel versus bevacizumab plus capecitabine as first-line treatment for HER2-negative metastatic breast cancer (TURANDOT): primary endpoint results of a randomised, open-label, non-inferiority, phase 3 trial. Lancet Oncol.

[b15] 15Alexis BC. Weekly paclitaxel plus bevacizumab versus docetaxel as second or third-line treatment in advanced non-squamous non-small cell lung cancer (NSCLC): Results from the phase Ⅲ study IFCT-1103 ULTIMATE. Oral abstract session presented at: 2016 ASCO Annual Meeting. 52^nd^ Annual Meeting of the American Society of Clinical Oncology; 2016 June 3-7; Chicago, America.

[16] Kurishima K, Watanabe H, Ishikawa H (2017). A retrospective study of docetaxel and bevacizumab as a second- or later-line chemotherapy for non-small cell lung cancer. Mol Clin Oncol.

[17] Hanna N, Shepherd FA, Fossella FV (2004). Randomized phase Ⅲ trial of pemetrexed versus docetaxel in patients with non-small-cell lung cancer previously treated with chemotherapy. J Clin Oncol.

[18] Besse B, Le Moulec S, Mazières J (2015). Bevacizumab in patients with nonsquamous non-small-cell lung cancer and asymptomatic, untreated brain metastases (BRAIN): A nonrandomized, phase Ⅱ study. Clin Cancer Res.

[19] Stefanou D, Stamatopoulou S, Sakellaropoulou A (2016). Bevacizumab, pemetrexed and carboplatin in first-line treatment of non-small cell lung cancer patients: Focus on patients with brain metastases. Oncol Lett.

[20] Adjei AA, Mandrekar SJ, Dy GK (2010). Phase Ⅱ trial of pemetrexed plus bevacizumab for second-line therapy of patients with advanced non-small-cell lung cancer: NCCTG and SWOG study N0426. J Clin Oncol.

[21] Herbst RS, Johoson DH, Mininberg E (2005). Phase Ⅰ/Ⅱ trial evaluating the anti-vascular endothelial growth factor monoclonal antibody bevacizumab in combination with the HER-1/epidermal growth factor receptor tyrosine kinase inhibitor erlotinib for patients with recurrent non-small-cell lung cancer. J Clin Oncol.

[22] Rosell R, Dafni U, Felip E (2017). Erlotinib and bevacizumab in patients with advanced non-small-cell lung cancer and activating *EGFR* mutations (BELIEF): an international, multicentre, single-arm, phase 2 trial. Lancet Respir Med.

[23] Seto T, Kato T, Nishio M (2014). Erlotinibalone or with bevacizumab as first-line therapy in patients with advanced non-squamousnon-small-cell lung cancer harbouring *EGFR* mutations (JO25567): an open-label, randomised, muticentre, phase 2 study. Lancet Oncol.

[24] Ardizzoni A, Tiseo M, Boni L (2012). Pemetrexed versus pemetrexed and carboplatin as second-line chemotherapy in advanced non-small-cell lung cancer: results of the GOIRC 02-2006 randomized phase Ⅱ study and pooled analysis with the NVALT7 Trial. J Clin Oncol.

